# The Undercommons of Childbirth and Their Abolitionist Ethic of Care. A Study into Obstetric Violence Among Mothers, Midwives (in Training), and Doulas

**DOI:** 10.1177/10778012231205591

**Published:** 2023-12-06

**Authors:** Rodante van der Waal, Inge van Nistelrooij, Carlo Leget

**Affiliations:** 1 36513 University for Humanistic Studies, Utrecht, The Netherlands

**Keywords:** obstetric violence, obstetric carcerality, obstetric abolition, birth, midwifery

## Abstract

Engaging in dialogue with critical mothers, midwives, midwives in training, and doulas in the Netherlands, this study furthers the theoretical understanding of both obstetric violence and the activist resistance against it. Obstetric violence is understood as part of a process of relational separation, leaving the pregnant person isolated. The activist resistance against it is consequently theorized as the abolitionist building of an alternative “otherworld” of radical relational care. The themes established are: (1) “institutionalized separation” with the subtheme's “expropriation,” “carcerality,” and “obstetric violence;” and (2) “undercommoning childbirth” with subthemes “fugitive planning,” “anarchic relationality,” and “obstetric abolition.”

## Introduction

Only a few years ago, obstetric violence was deemed to be an unnecessarily provocative term which would guarantee heated reactions from midwives, doctors, hospital management, and policymakers. Fortunately, during the last couple of years, the problematization of obstetric violence has become more mainstream. With the growth of consciousness around obstetric violence, however, we tend to hear more about the traumatic birth experiences of victims, and less about the knowledge that those affected, such as mothers, doulas, and midwives (in training), have developed–both in the form of critique and in the form of care alternatives. In the study of obstetric violence, or what is often euphemistically called *respectful maternity care*, we, as care workers, academics, and policy makers, must be careful not to instrumentalize the experiences of traumatic birth for the reform of obstetric services in the way we deem it best and thereby reproduce an active–passive dichotomy on the part of pregnant people as victims on the one hand, and the reforming institutions of healthcare and science as agents of change on the other. For most activists, calling out obstetric violence always went hand in hand with the radical demand for, and practice of, relational continuity of care, oftentimes preferably outside of the jurisdiction of the obstetric institution. But currently, the proposition for obstetric reforms has gotten the overhand over a more radical reimagination of reproductive care. As a consequence, the general difficulty and disappointment that comes with the attempt to reform big and powerful institutions can lead to the impression that obstetric violence is a problem that is near impossible to tackle. As such, the attempted reform of the obstetric institution by the institution itself, disguises the many solutions to obstetric violence already offered and practiced by mothers, midwives, and activists, such as mutual aid and radical care outside of the obstetric institution–most often coming down to relational continuity of autonomous midwifery and doula care.

In this paper, we aim to take seriously the knowledge developed by engaged and activist mothers, midwives, midwives in training, and doulas on both the nature of obstetric violence *and* on alternative ways of care. We do so by pairing the views of the participants to two critical traditions of thought; care ethics, and abolitionist thought as articulated by the Black radical tradition. Both traditions have developed a body of thought on a multiplicity of patterns of racist and misogynous violence. Adhering to a “practicalist conception of truth,” this paper engages with the moral and epistemic standpoint of critical and/or activist mothers, midwives, midwives in training, and doulas in the Netherlands, with regards to the problem of obstetric violence *and* its solutions, aiming to present their thought, analysis, actions, and resistance, rather than merely their experiences of victimhood (Ruddick, 1989, p. 13). As such, this paper is written from the methodological perspective of the “rear guard intellectual” and “scholar-activist”, doing intellectual labour in the backseat of the movement, i.e., in support of the knowledge and actions already being developed by the movement itself, documenting, clarifying, and theoretically strengthening its position (de Sousa Santos, 2018). As such, this study is a contribution of coalition and solidarity that will hopefully be useful to take our radical care and struggle further.

Although multiple definitions exist, we understand obstetric violence to be the institutionalized appropriation and violation of pregnant people's bodies, expropriating them of their self-determinacy, autonomy, responsibility, community, the right to physically and emotionally safe care, and the choice to birth or not birth their children in the way that they think is best (Van der Waal, Mayra et al., 2022; Van der Waal, Mitchell et al., 2021). Examples of obstetric violence in the Netherlands are procedures done without consent, such as vaginal examinations and episiotomies, verbal and physical abuse and mistreatment, and epistemic injustice (Van der Pijl, Hollander, et al., 2020; Van der Pijl, Klein Essink, et al., 2023; Van der Pijl, Verhoeven, et al., 2022; Van der Waal et al., 2021; Van Hassel et al., 2022). In the Netherlands, 54% of parents indicate that they were subjected to one of more forms of mistreatment and abuse, of which 20% to physical violence (Van der Pijl et al., 2022). Of the people who get an episiotomy during birth, 42% indicates that they were not asked for consent (Van der Pijl et al., 2023). Another 47% of the people who got medication states that they were not asked for consent for its administration (Van der Pijl et al., 2023). On top of that, people report getting episiotomies, vaginal examinations, and medication under their explicit refusal: 40% of those who refused an episiotomy got one anyway, and the same counts for 40% of those who refused a vaginal examination, and 60% of those who refused medication. (Van der Pijl et al., 2023). While health care workers within the obstetric institution and their allies (see for instance Lappeman & Swartz, 2021, and see Chadwick, 2023 for her response to this article) still tend to react defensively to the term *obstetric violence*, and while many people still do not know this rather invisible form of gender-based violence–maybe because it is one of those kinds of violence that is too public, too institutionalized, too normalized, to be noticed—the problem of obstetric violence is put on the agenda in maternity care ever since the publication of various major studies in high ranking journals (for instance, Castro & Frías, 2020; Sadler et al., 2016), and the United Nations report (Simonovic, 2019). By now, the meticulous analysis of the racist and gender-based nature of this form of violence, both philosophical (for instance, Cohen Shabot, 2016; Kingma, 2015, 2021; Villarmea, 2020), sociological (for instance Chadwick, 2018; Smith-Oka et al., 2022), anthropological (for instance, Davis, 2019), juridical (for instance, Pickles & Herring, 2019) and epidemiological (for instance, Bohren et al., 2015; Sadler et al., 2016), are being used to battle obstetric violence on juridical and policy-making levels.

Obstetric violence is an activist term, coined in South America, a “struggle concept” (Chadwick, 2021). But while the voices of the activist mothers, midwives, and doulas have been heard where it comes to the recognition of the existence of obstetric violence, their voices tend to remain lost in academic, juridical, and policy-making discourse when it comes to their resistance to, and solutions for, obstetric violence. In struggle, critique always goes hand in hand with the fight for a better world, and with a vision, often already put into practice, of how this world should be. Aiming to take serious that obstetric violence is a struggle concept, and wanting to understand what the struggle is *for* as much as what it is against, we engage in an analytic dialogue with mothers, doulas, and midwives about what they believe is reproductive and birth justice, understanding their practice to be the moral expression of what they conceive of as justice (Walker, 2007; for an introduction into the Black feminist concept Reproductive Justice see Ross & Sollinger, 2017 and the SisterSong Women of Color Reproductive Justice Collective). This approach confers that mothers, doulas, and midwives are not only people with experiences that we can turn into knowledge which can then be used in various ways for research and policy, but that they are themselves agents of knowledge, praxis and change, who are already planning and organizing alternatives to obstetric care. Harney and Moten (2013) make a crucial distinction between policy and planning, whereby policy captures all interventions done at a managerial level out of institutional power, such as the writing of guidelines, the issuing of reports, the setting of goals and making of strategies, while planning contains the direct, grassroots action that people are already undertaking through mutual aid to create a different world through praxis; the difference between writing a guideline on respectful maternity care, and opening an alternative practice where accessible, relational, personalized continuity of care is guaranteed. In a world where institutions and policy are hegemonic, the planning of care and activism tends to stay invisible. This is a problem, since solutions mostly sprout from active on ground resistance and reimagination—indeed, from their unique moral and epistemic standpoints. The dual aim of this paper is hence to dissect, affirm, and theoretically further develop what activists, doulas, and autonomous midwives know about obstetric violence—taking their critique seriously as agents of knowledge, *and* to understand better the alternative practice that they have been planning in terms of how this practice is a resistance to, and solution for, obstetric violence.

What emerges from this study, then, is that obstetric violence should not be understood as a stand-alone problem that is easily circumscribed, but as part of a bigger logic inherent to the obstetric institution that separates relationality. The intertwined expropriation of maternal subjectivity, the carcerality of the obstetric institution, characterized by captivity and punishment, and obstetric violence leads to the isolation of the pregnant person. Since the participants do not see obstetric violence as a stand-alone problem, or even as something that you could cut out of the obstetric institution, as it is so intertwined with a more fundamental logic of separation inherent to the institution, their resistance to obstetric violence is neither characterized by reform, nor can it be understood on a level of policy. Instead, their resistance must be viewed as a form of planning of alternative forms of care that takes them outside of the obstetric institution. When we zoom in on these practices of planning, what emerges is an almost invisible but vast “undercommons” of care for childbirth (Harney & Moten, 2013, 2022) that is expressive of an abolitionist ethic of care: An ethic of care that is not trying to reform the obstetric institution, but that has a vision of its abolition–and with that, a vision of a different world altogether.

Below, we will first concisely discuss our theoretical framework and positionality, after which we will proceed to extensively elaborate on our methodology. Then, we will present the results of the empirical study, consisting of two main themes representing the critique of obstetric violence on the one hand, and the specific form of resistance to it on the other: (1) *institutionalized separation*, consisting of expropriation, carcerality and obstetric violence, and (2) *the undercommons of childbirth*, consisting of fugitive planning, anarchic relationality, and obstetric abolition. Thereafter, we will discuss that the primary way of struggle against obstetric violence of the mothers, midwives, midwives in training, and doulas in this study, is through a praxis of an abolitionist ethic of care which resists the isolation of mothers and midwives inherent in the obstetric institution.

## Theoretical Framework and Positionality

### Theoretical Framework: Care Ethics, Abolitionism, and Critical Midwifery Studies

Care ethics is a feminist ethics departing from the idea that we are all relationally connected through care and responsibility, and therefore vulnerable in our mutual dependency (Tronto, 1993, 2013). Consequently, the mothers, doulas, and midwives in this study are understood to be always already relationally embedded in a practice, and an ethics, of care. The practice of care that they engage in, is taken as the direct source of the various, multiple, and always becoming moral and epistemic standpoint that is being studied. By taking the relationality of midwifery, including mothers, doulas, and midwives in training, as its relational moral and epistemic standpoint, this study is also situated in the emerging field of critical midwifery studies (CMS), which understands midwifery to be a marginalized standpoint through which we can “study upwards” the intersections of oppression present in reproductive care (CMS Collective, 2022; Mohanty, 2003). CMS believes in the potential of midwifery to better neonatal and maternal health outcomes, something that is consistently proved by midwifery scholars, as well as to reduce obstetric violence and obstetric racism, but only if midwifery can incorporate critical theory such as intersectional feminism, the Black radical tradition, and decolonial theory (ibid.). We aim to contribute to the development of CMS by taking both care ethics (explicitly Tronto, 1993, 2013; Walker, 2007) as well as Black abolitionist scholarship and activism (explicitly Bey, 2020, 2022; Harney & Moten, 2013, 2022) as a theoretical framework to understand better what midwives and doulas know about obstetric violence and how they struggle against it.

All participants in this study have a uterus, have experienced obstetric violence, and are of diverse professional, socio-economic, sexual, gender, and cultural identities and backgrounds. Some participants identify as Black, some as white, some as people of colour. Some are born in the Netherlands, and others identify as having a migration background. All participants have extensive knowledge of the subject additional to their own experiences, obtained through either study, activism, or care. Following standpoint theory, their identities and experiences have familiarized them with structures of oppression and gave them insight into knowledge that would have otherwise remained unfamiliar to them (Harding, 2004). Next to a common identity, a certain profession, such as midwifery, can also be considered the basis of unique knowledge. Informed by critical midwifery studies (CMS Collective, 2022) as well as care ethics, we locate the practice of midwifery as the moral and epistemic standpoint studied here, but only in so far as midwifery is understood to be a relationality including pregnant people and others supporting the labouring person. This research is hence not focused on either midwives, or mothers, or activists, but on the relational *coalition* between these different subjects. In other words, the standpoint that is studied is that of midwifery, but only in so far midwifery can be understood—or must wish, and fight, to be understood as such—as a bundle of relationality between people giving birth and people caring for people giving birth; a sociality that is irreducible to demarcated subjectivities. A sociality, if you will, that always thinks, practices, and struggles in relation, and that resists to be fully individualized or professionalized.

### Positionality

The main author, Rodante van der Waal (she/they), writes this paper as a white middle class PhD-candidate from Amsterdam and as an independent midwife that is part of the standpoint being studied here. The second author, Inge van Nistelrooij (she/her), is a white middle class senior academic, and mother of three daughters. Her standpoint as a mother was enabled by the obstetric institution that provided both life-sustaining and harmful practices. The third author, Carlo Leget (he/him), is a white middle class senior academic, father of three children, one of whom recently gave birth herself. He was closely involved in the home birth of his three children. All authors have clear ties to the subject at hand. We believe that by acknowledging the borders of our positionality and by staying faithful to our own subjectivity and the particularity of this research and our identities, we will be able to say something about widespread intersecting structures of oppression in obstetric care while staying acutely aware of the limitations of this study caused by the limitations of our identities and positions (Mohanty, 2003).

## Methods

### Research Design

The study is designed according to the insights of both standpoint theory which regards experiences of marginalized people as a source of knowledge that often remains unrecognized in more traditional and objectivist research methods (Harding, 2004), and the insights of care ethics, which holds that theory and empirical data are always constituted in a dialectic and cannot be objectively separated (Leget et al., 2017). To facilitate a process of study that was able to both engage with the standpoint of the participants, and further the theoretical understanding of this standpoint through the interaction of insights gained by theoretical study and insights gained by empirical study, the study was designed according to the method of responsive evaluation (Abma & Widdershoven, 2005), in a version specifically adapted to care ethics (Visse et al., 2015). Responsive evaluation is a democratic and dialogical method, offering room for interaction and exchange of experiences and ideas among participants and researchers. It consists of five steps: (1) creating social conditions; (2) eliciting experiences from different stakeholders; (3) homogeneous focus groups; (4) heterogeneous focus groups; (5) drawing up conclusions and recommendations. All five steps were followed, please see paragraph 3.3 for the details on step 2, 3, 4 and 5. In line with care ethics, our theoretical framework was only developed *during* the phases of the empirical research, and in dialogue with the participants.

### Participants and Sampling

Thirty-one participants were recruited by the first author of this paper; ten mothers, eleven midwives, five doulas and five midwives in training. Most of the birth workers involved are also mothers, many of whom have experiences of obstetric violence during their own pregnancy and childbirth. The main author contacted people through their personal network, as well as through the activist organization the Birth Movement (*Geboortebeweging*). Participants were also recruited via snowball recruitment. Sample criteria were extensive knowledge (either scholarly, activist, or embodied) of obstetric violence additionally to personal experiences with it, and engagement with either activism or alternative forms of care. This means that all participants were critical (ranging from quite to very critical) of Dutch maternity care, and that they have experience with, and ideas on, how care for birth can be better. We have hence specifically selected a group of participants that is almost certainly more critical of Dutch maternity care than an average prospective parent, in order to engage with the analytical knowledge on the nature of obstetric violence from people in the field. To be able to include a breadth of perspectives, analyses, and practices, within this critical branch of people, attention was paid to establishing a diverse group of participants in terms of both identity (i.e., with regards to gender, sexuality, racialization, and class) and practices, with some engaging more in direct activism, others in reading and study, others in art, and others in alternative forms of care, but all relating to obstetric violence and the resistance to it.

### Data Collection

Following the method of responsive evaluation (Abma & Widdershoven, 2005) adapted to care ethics (Visse et al., 2015), data were collected in three rounds: individual interviews, homogenous focus groups, and heterogenous focus groups. Interviews were conducted by the first author in 2020. Because of the COVID-19 pandemic, almost all of them were online. They all lasted a median of a bit over two hours and were in-depth interviews. They were minimally semi-structured based on familiarization with the interviewee's thought as expressed in previous conversations, publications, Facebook discussions, etc. We used minor personalized interview guides with some themes and questions based on this prior familiarization to lift the conversation more quickly to an analytical level. There were no additional external themes added to the topic list. Open, non-directive formulations that were consistent with the interviewee's own vocabulary were used. Notes were made during the interviews. The interviews were recorded, anonymized, and transcribed ad verbatim. Two were wrongly recorded and got lost, but the notes were still used. After the first round of individual interviews, they were preliminary analyzed by the first author, and a thematic analysis (TA) (Braun & Clarke, 2006) with some extra theoretical interpretation based on our developing theoretical framework was sent to the participants. The participants were given the opportunity to give feedback in writing or during the following focus groups.

Homogenous focus groups were conducted at the end 2020 and beginning of 2021 (Abma & Widdershoven, 2005; Visse et al., 2015). Because it was difficult to meet all at the same time, multiple groups were formed, six in total (twice four midwives; once five doulas; once four midwives in training; once three, and once four mothers). The focus groups were semi-structured with a topic list based on the analysis of the individual interviews. They lasted a median of a bit under three hours. They were all done online, recorded, anonymized, and transcribed ad verbatim. The homogenous focus groups were then again preliminary analyzed, and this TA (Braun & Clarke, 2006) with an extra further theoretical interpretation based on our developing theoretical framework, was again sent to the participants for feedback. The participants were given the opportunity to give feedback in writing or during the following focus groups.

Heterogenous focus groups were conducted in 2021 (Abma & Widdershoven, 2005). Three heterogenous focus groups were done since it was difficult to get more people together on the same date (twice with five participants, and once with six). The focus groups were semi-structured with a topic list based on the analysis of the homogenous focus groups, and started with a reimagination exercise wherein all participants were asked to write down their preferred form of birth care if everything would be possible and then to read it out loud, after which they wrote down a reaction to each other, and read this out loud again. Afterwards, a free-floating discussion took place. All focus groups lasted a median of a bit over three hours. They were online, recorded, anonymized, and transcribed ad verbatim.

### Data Analysis

A new TA was conducted after the phases of data collection by the first author through Atlas.ti under supervision of the other authors (Braun & Clarke, 2006). TA has five phases before drawing up the results: (1) familiarizing yourself with the data; (2) generating initial codes; (3) searching for themes; (4) reviewing themes; and (5) defining and naming themes (Braun & Clarke, 2006). All phases were done and discussed, together with the codes, code tree and themes, with all authors. TA differentiates between an inductive and a theoretical approach. The theoretical approach is informed by the theoretical lens and research question of the authors, while the inductive approach establishes the research question bottom up through the coding process. Since care ethics recognizes that one can never separate empirical findings from theoretical insights (Leget et al., 2017), we chose the theoretical approach which was, in our case, further informed by care ethics (explicitly Tronto, 1993, 2013; Walker, 2007) as well as by abolitionist theory (explicitly Bey, 2020, 2022; Harney & Moten, 2013, 2022). The theoretical framework was only developed *during* the different phases of data collection, and was thus informed by the dialogues with the participants. The names of the themes and subthemes attest to the theoretical framework that we developed congruently with the empirical study, as they aim to grasp most closely what the participants have said, while at the same time referring to concepts that originate from the above theoretical traditions. For instance, although the participants did not use the, quite theoretical, term “expropriation” themselves, many of them did describe the experience of expropriation and understood this to be one of the causes of obstetric violence. The theoretical name of the theme is here used to further our analytical understanding of what obstetric violence is, based on an analytical engagement with the standpoint of the participants, rather than solely describe their experiences. The same counts for the subtheme “carcerality.” Although the participants did not use the word carcerality, they did use the words “captivity” and “punishment,” and many of them understood these terms to re-enforce each other and be co-constitutive of an overall feeling of unfreedom and disciplination. The term carcerality captures, in our understanding, the knowledge that the participants were communicating, combined with our theoretical study of the power structures inherent in institutions. The themes hence closely grasp the participants' standpoint while at the same time establishing a connection to theoretical thought—this is, according to de Sousa Santos (2018), the added value of the work of scholars in activist movements. In sum, the subthemes reflect the result of the analytical dialogue with the participants through the phases of care ethical responsive evaluation wherein empirical and theoretical research are used to guide and develop one another. Since this research takes place on a level of dialogical thought, analysis, and knowledge, rather than being merely descriptive, we consider the choice of the theoretical approach to TA as a further thinking along with the participants that deepens understanding, knowledge, and insights (Walker, 2007).

### Ethical Considerations

This research was evaluated and approved by the ETC of the University for Humanistic Studies in 2020. The METC of the University of Utrecht decided that the Dutch Medical Research Involving Human Subject Act (WMO) did not apply, as participants were not patients but mentally competent citizens, and participants were not subjected to treatment nor required to follow a certain behavioral strategy as referred to in the WMO (art.1b). All participants were given an information sheet prior to the study and room for questions at the beginning of the individual interviews. Privacy details were discussed and their right to withdraw at any moment was made explicit. They all gave written informed consent to their participation in the study and most for the anonymized publication of the interviews and focus groups in the DANS archives.

## Results

Two main themes and various subthemes were established. The first is “institutionalized separation” with the subtheme's “expropriation,” “carcerality,” and “obstetric violence.” The second theme is “undercommoning childbirth” with subthemes “fugitive planning,” “anarchic relationality,” and “obstetric abolition.”

### Institutionalized Separation

The mothers describe an overall sense of isolation within the obstetric system, as a result of a severance of two relations: that between the mother and her child, and that between the mother and her community of care. The mothers construe how a felt separation between mother and child takes place when care workers take over the responsibility over the baby, thereby negating maternal responsibility and knowledge that is essential to their mother–child relation. Consequently, this violation of trust, in a combination with feeling objectified, violated, captive, and expropriated, is described as a severance of the relationality between the mother and her community of care, resulting in the experience of isolation. The theme institutionalized separation hence consists of three subthemes: (1) expropriation, where the responsibility over the baby and the autonomy over the maternal body are expropriated; (2) carcerality, consisting of a feeling of isolation and captivity of both the mothers and birth workers combined with the threat of punishment; and (3) obstetric violence, consisting of continuous and overlapping experiences of violence. “Expropriation” is a term borrowed from Federici (2007), and “carcerality” is a term borrowed from Bey (2022).

#### Expropriation

Most mothers in this study understand themselves to have intuitive or bodily knowledge on how to birth, on how to be in a relation of care and responsibility with their child when pregnant, and as embedded in a community of care and responsibility. Mothers felt actively expropriated by the obstetric system of their capacity to birth:

A lot of people in the hospital told me, “It's my job to keep the baby safe.” But I didn’t see that as their job. I thought their job was to help me birth. It's their job to keep me safe, it's my job to keep the baby safe. […] But that was not very—actually, that was never mentioned. This felt really lonely. I felt suddenly I didn't speak the language anymore. In books, in writing, I found people who shared these opinions but they weren’t with me; they were not having coffee with me, were not here to be my midwife and were also not my partner. I know other people felt the way I felt but I didn't find them. (Mother 1)

In the quote above, the hospital staff takes responsibility over the baby rather than that they take care of the mother and let the mother take responsibility over her child, causing the mother to experience isolation, and loss of autonomy over herself and her child. While mothers expected to gain in care and relationality when entering the obstetric system, they instead felt more and more devoid of what they understood to be care the further they get into the obstetric institution, as if the institution was actively taking something away from them:

Everything went fine until the staff in the hospital realized that I was already pushing, […] I was laboring, and my husband was with me, and we had a really good… we had rhythm, and a little ritual, equal, and it was good. But then, at some point somebody heard all the sounds coming and recognized this is a woman who is bearing down, and at that moment everybody rushed into the room. A lot of team, a big team rushed into the room, they got me to move onto another bed, and pushed me into the delivery room, and from that moment on it was about push, push, push, and I felt very bad. […] I became like a procedure, it wasn’t about me, […] Yeah, I think from that moment on I didn’t feel anymore it was, it wasn’t my experience, it wasn’t about me, it was about the baby coming out […] it wasn’t anymore mine […] it was delegated to all these others. (Mother 3)

Rather than going further into the relation and rhythm of care that this mother already established with her husband, which felt good, wherein she knew what to do, and wherein they could experience the birth together, the staff took over, appropriating her laboring body, and, quite literally, expropriating both parents from their autonomy, experience, relationality, knowledge of, responsibility over, and care for birthing their child. Consequently, the mother is isolated from both the relation to her child as well as her community of care, in this case her husband.

Mothers experience expropriation on multiple levels: an expropriation of their body which no longer feels as their own; an expropriation of their experiences, wherein they either do not matter or they have no control over them; an existential expropriation wherein they do not get the freedom to experience birth as something more than a clinical and biological experience; and expropriation of their relation with their child and the community of care wherein they were embedded. Some participants regard these layers of expropriation, to be the core of obstetric violence:

The core of obstetric violence in my view lies in handing over every aspect of your functioning to a professional. We do not seem to understand how disempowering that really is. (Midwife 10)

#### Carcerality

Carcerality is chosen as the name for this subtheme to capture an intertwined and mutually constitutive experience of both captivity, described by both mothers and birth workers, and a fear of punishment, again described by both mothers and birth workers. Together, the experience of captivity and the fear of punishment, amount to what can be called a carceral tendency within the obstetric system. In the same movement wherein expropriation (discussed in subtheme 1.1) takes place, participants describe an appropriation of the mother's body:

I was on my knees giving birth in the bathroom and she came in and she said, “Get on the bed! I must examine you.” And I said, almost begging, I asked her, “Can’t I stay here?” And she says, “No. I really have to examine you.” She looked at me and she spoke to me as if I were a difficult child who wouldn’t listen and said, “Come on, I really have to feel the dilation.” And then, I had to go to the birth clinic, we raced there, and within half an hour […] my child was born. And afterwards I thought that I might as well have just stayed in that bathroom and given birth to my child in a way that felt good to me, but she wanted to examine me and then she really wanted to hand me over to the hospital, because there was also another delivery going on and she was really persuading me […] I was an inconvenience. I was being difficult. And it wasn't really my birth anymore. And I thought afterwards: what if it had been my birth? I would have loved to stay in that bathroom and just have my kid there. (Midwife in training 2)

In the quote above, it is described by a midwife in training how she, when she gave birth herself, had an unnecessary and unconsented vaginal examination and was afterwards being transport to the hospital without consent. In the Netherlands this is a clear violation of choice, bodily autonomy, and freedom of movement, since homebirth is an integral part of the Dutch obstetric system. She describes how her body and birth no longer belonged to her, and how the way in which she was birthing her child, which went well, felt good, and was safe, was interrupted by the midwife who was taking charge of her body and her labor. She describes how she was brought to the hospital against her will, and how she and her birth were captured by the obstetric system in the same movement as she was expropriated of responsibility, autonomy, and self-determination. Reflective of an expectation of punishment, like with difficult children, she describes how the midwife related to her as "being difficult."

For both mothers, midwives, midwives in training, and doulas, the institution feels like a system wherein they get captured through disciplination, governance, protocols, and punishment:

It's a form of captivity to work in this system. How can you empower or make space for women when you have no space or power yourself? (Midwife in training 4)

[The care] had a very controlling aspect, they assumed that they had to act like a detective to find out what had already gone wrong with the pregnancy or would go wrong with the pregnancy. Yes, that was the terrible thing about it. (Mother 2)

This mother had a healthy pregnancy, but wanted to be cared for in a different way, which was met with punishment in the form of suspicion, controlling behavior, and shroud waving. Participants also point towards a threat of punishment that disciplines pregnant people as well as care workers, before they do anything out of the ordinary:

If I comply, then I cannot be punished. [They] are saying: you are either with us, and if you’re not, then you’re on your own. And then comes the blaming and the shaming. (Mother 9)

When I look back to when I would always stick with the protocols and all the time refer to the obstetrician, well that was not because I thought that it was the best thing for mother and child. […] It was because… because I don't know how it ends either. And if it doesn't end well, I hang. I hang. (Midwife 7)

Care workers fear legal repercussions as well as social ones. They express that this fear disciplines them to be complicit to the institution, which contributes to the overall theme “institutionalized separation” as it separates them relationally from the mother, being bound stronger to the institution than to the people they care for. Even though being a doula and a midwife in primary care are still independent professions in the Netherlands, both describe being appropriated by the obstetric system to function as part of the logistics of the system, alongsides the expropriation of their professional subjectivity. Similar to the mothers, they note that obstetric care circumscribes and confines them and punishes them when they are seen as difficult. For midwives and doulas this mostly leads to a sense of forced complicity to the carcerality of the institution. For instance when people's bodies are “handled” without consent, while the midwives and doulas do not dare to truly intervene:

I think it was a Moroccan woman […] And at one point, she had to pee. But she wasn’t allowed to get off EFM. And then they wanted to give her a catheter. But she didn’t want one. And then I said: she doesn't have to get one, she can just go to the toilet. But no, she really wasn't allowed to get off the bed. And then she was put on top of the bed on a birthing stool in the middle of the room to pee, on which she sat with her bare buttocks. I felt that it was not ok for her, with those two strange men also present, two male interns, […] it was very naked for her. (Midwife 7)

Scenes like the one above describe that laboring people can experience a captvity within obstetrics that feels like punishment. This captivity simultaneously severs the relationality of the mother with her community of care, and takes away her authority and responsibility over and her child. At the same time, it perpetuates instances of violence and punishment on all levels of care, even when the care workers, like in the quote above, did not seem to have the intention of punishing or violating the birthing mother. This logic of captivity and punishment, is what we have termed carcerality. The participants understand the carcerality of the institution mostly to be a consequence of the prioritization of the unborn child above all else. We could understand this prioritization as a first principle that commands the rest of the care:

The ultimate consequence of saving the child above all else, is that they will drag the mother in a military manner to the hospital if necessary. (Mother 6)

#### Obstetric violence

Most participants view violence within obstetric care as institutionalized violence, rather than as violence coming from one specific person. They furthermore understand violence to be intertwined with the expropriation of subjectivity of mothers, midwives, and doulas, as well as with a logic of captivity and punishment that we have termed carcerality, described above. This means that most of the participants do not regard obstetric violence as a stand-alone problem that is easily circumscribed, but as part of a bigger problem, namely a logic inherent to the obstetric institution that primarily separates relations. Ultimately, obstetric violence is thus only one of the subthemes that co-constitute the experience of isolation central to obstetric violence due to the severance of relationality, and is seen more as a necessary part of, rather than an incongruence within, the obstetric institution.

The most listed occurances of violence in obstetrics in this study are: (1) obstetric racism; (2) epistemic injustice, mainly playing the dead baby card; (3) physical violence, consisting of interventions without consent; (4) penetrative violence, that is, violence linked to, or reminiscent of, rape or sexual assault; and (5) unconsented and/or unwarranted, and/or unwanted vaginal examinations. In the quote from a midwife in training below, all five of these forms of violence are present in a situation wherein a vaginal examination is being done. This quote demonstrates how different forms of obstetric violence overlap with each other, and how violence is part of the expropriation and carcerality described in the subthemes above:

She was so scared, and I said to the midwife, “I know it's evening, but can't we get an interpreter to translate?” […] But the midwife said, “You know, just do it”. And then I said, “But she doesn't understand it.” You know, I'm not going to just put my fingers in someone, while someone doesn't understand, I think just really that that is a form of rape. That's real, you know. And I just don't want to do that. […] I notice that when people are foreign and do not speak Dutch, they also use the opportunity to just do it, to let the student practice more. […] People are side-lined. My sister-in-law told me about her birth that “someone else came in again, didn’t even say anything and just stuck their fingers in me.” [..] I am familiar with sexual violence. So, for me it's extra difficult if I see midwives do that, if they go in with their fingers while someone screams: “No, stop! What are you doing? Stop!” Yes, for me, that is even more difficult, to…. Yes. (Midwife in training 3)

In the quote above, it becomes clear how tricky it is to differentiate between different forms of obstetric violence, and to differentiate obstetric violence from other structures of oppression within the obstetric system. Multiple instances of violence are intertwined and point toward a problem of widespread cultural and institutional violence characterized by expropriation and carcerality. First and foremost, obstetric racism is present in this quote. There is no interpreter, the laboring person does not understand what is going on, and the midwife in training says that people who do not speak Dutch are used to practice on more, which is a classic form of medical racism and medical apartheid. Second, we see the presence of epistemic injustice. The mother does not understand what is going on and her lack of understanding is used to the benefit of the institution. Third, there is physical violence in the form of a medical intervention without consent. Fourth, there is penetrative violence; a vaginal exam about to happen with neither consent nor understanding, and the midwife in training refers to other instances of penetrative violence. Here, it also becomes clear how mother and midwife cannot fully be separated as different standpoints. While we do not know if the unconsented vaginal examination was reminiscent of rape to the labouring person, it was for the midwife in training. The obstetric violence here, thus also concerns her as it was triggering of past trauma. And fifth, it is a case of an unwarranted and unwanted vaginal examination. Clearly it was unconsented, but was also unwarranted since the only indication for the extra student exam was for the student to practice.

The above quote also shows how obstetric violence intertwines with the expropriation of the mother's knowledge and autonomy, and how the violence is part of the carcerality of the system, as it amounts to the captivity of the mother's body by taking charge of it for practice, and the experience of punishment which is not only present in the other mothers screaming “no,” but also in the felt threat of punishment to the student midwife if she does not comply. It becomes visible in this quote how the subthemes of expropriation, carcerality, and even violence, all also count for the midwife in training who is held captive in the push to complicity with something what she experiences as, and has in the past experienced as, sexual violence. It thereby shows as well how not only mothers, but also midwives and midwives in training are expropriated, made complicit to the obstetric institution through its carcerality of punishment and capture, and pressured to participate in its violence—all contributing to the overall theme of institutionalized separation that constitutes maternal isolation.

### The Undercommons of Childbirth

Rather than fighting the institution, the midwives, mothers, and doulas in this study mostly opted to “flee” from it, planning different alternative practices themselves. They reappropriated care outside of the obstetric institution, taking their care, birth, and pregnancy into their own hands. Considering all the practices that they have enabled or that they participate in, a grand underground landscape of care for childbirth becomes visible that exists either fully outside of the institution or holds space within the institution. This landscape, that relies on relations, collaborations, unofficial networks, and mutual aid, could be characterized as an undercommons. If the obstetric institution is the national commons of reproductive care, the invisible networks and resources of care that do exist outside of the obstetric institution, can be understood as an invisible underground of care. These undercommons are organized in various ways; through doula-communities, through a big group of alternative midwives sharing knowledge and asking each other for help, or, for instance, through the activist movement the Birth Movement (*Geboortebeweging*), that many of the participants are affiliated with. The Birth Movement has a phone you can call 24/7, a very active Facebook page where mothers and birth workers can ask for help, and that is a resource of knowledge on birth, and a network of many midwives and doulas that provide alternative and respectful care. These undercommons also extend to *within* the obstetric institution through coalitions built with obstetricians and gynecologists, through which the public resources of the obstetric institution can be used outside of its protocols. The “undercommoning” is done by mothers and midwives through fugitive planning, the reconstruction of relationality in an anarchic way, and a vision of abolition. In the elaboration of the theme below, the undercommons are hence understood as constituted by three subthemes: (1) fugitive planning; (2) anarchic relationality; and (3) obstetric abolition. “Undercommons” and “fugitive planning” are terms borrowed from Harney and Moten (2013).

#### Fugitive planning

Many, almost all, mothers were determined to take matters into their own hands after the birth of their first child wherein they had experienced obstetric violence. Refusing the further expropriation of their relation to their child as well as to their community of care, mothers planned their second birth fugitively, searching for the relationality that was denied to them during their first birth, and found independent alternative midwives outside of the obstetric institution. Their “flight” made their birth an “underground action” and they wanted to protect it as such:

It is an opting out, the building of a refuge to flee away from, the making possible of a place from where you can go elsewhere. […] I described it myself, to a friend of mine who was also pregnant, we always said to each other: we must stay under the radar. We shouldn't be in the picture. This is an underground action. Once they see you, you can't get away, so you must stay under the radar. […] Stay away from the hospital, that's what we did. (Mother 4)

The midwives whom the mothers found were either working mostly outside of the institution already, or they started to plan fugitively along with the mothers:

So, they [the mothers] realize that the world is much more beautiful and bigger when they look outside [the institution]. And at some point, I made the fundamental decision: if someone wants it, I'll go with it. […] And if it doesn't feel safe, I'm going to look if I could do it with someone who has more experience. […] In principle, anything is possible. […] It means that all protocols and guidelines will be placed in a completely different light. And when I made that decision, I stopped working in regular care. (Midwife 3)

Mothers either first went back to their old midwifery practice or hospital with new demands but were refused care, or they refused the care offered by the institution themselves and started looking for alternative care when they were pregnant:

And the second time, I was on to them […] I absolutely did not want to start in the hospital for the second time. With a VBAC, it's very difficult, but I said, I don't do it, forget it. I just straight out refuse that. I made a whole scene and then found a construction with a doctor and a midwife [outside of the hospital] who would allow me to birth at home. (Mother 6)

Some people even only got pregnant again after they had built a relationship with an alternative midwife that would accompany their birth outside of the institution. In contradiction to free birthers, all the mothers in this study did want care; it was the reappropriation of a community of care that mattered most to them. Their fugitive planning was not only a planning towards autonomy or merely freedom from oppression, but a search for care that can only be found in community. They describe it as a resistance to the institutionalized double separation (between mother and child, and between mother and her community of care) described in theme one. Both mothers and midwives hence plan alternative forms of care *together* where they can be in safe relation with each other and wherein birth workers are not forced to be complicit in expropriation, carcerality or violence. They do this either fully outside the institution or in collaboration with birth workers within the obstetric institution. As such, mothers organized the most complex medical care cases responsibly and, in their opinion, more safely, than they could have if they had followed the protocols of the obstetric institution. They pushed midwives further, made new connections, and got involved in birth activism. As such, their fugitive planning contributes to the existence and growth of an undercommons of childbirth care. The existence of these undercommons gives hope to most participants, especially to midwives in training, keeping the possibility open for them to also plan fugitively toward them:

Knowing that there are other midwives and a growing group of birth workers that practice the way that I want, and that they will also welcome me, that they reach out for me, that they are there for me, that helps me a lot. If they hadn't been there, you would have a future of only obstetrics that you really don't agree with. Then, I would have to go in a completely different direction on my own. I would probably have stopped then. Yes, I think so. (Midwife in training 5)

The planning of mothers, midwives, and doulas, thus also makes a future undercommons of birth possible for a next generation of midwives, functioning as a point on the horizon. As such, it also re-establishes a relationality between birth workers themselves, characterized by mutual aid rather than enmity and captivity, and between birth workers and the people they will care for: they now have a prospect on a form of relationality that they believe in, rather than seeing no other option than having to become complicit in a violent form of care.

#### Anarchic relationality

But what makes the relationality of care in the undercommons so different than the severed relationality within the institution? Instead of a relationality that is limited and inhibited by carcerality and expropriation, and the hierarchy and authority that come with it, the relationality in the undercommons of midwifery care is understood by the participants as fundamentally non-hierarchical in terms of professional authority but also in terms of principles: there is no longer a first principle, like saving the life of the child at all costs, that justifies the carcerality or dogmas of the rest of the system. We could hence qualify this relationality as anarchic, in the literal meaning of the word as an-arche, that is, without first principle. There is nothing expect what develops in the relation itself that equals an order, a hierarchy, or a rule. This anarchic quality of the relation changes the definition of care from something that is established in a certain way in protocols, to needs which can only come forth out of the relation itself:

If nothing asks for attention, attention does not need to go there. Then, all attention is with mother and baby and I’m out of the picture. (Midwife 3)

Anarchic relationality does not mean that the care is not organized or that it is chaos, rather the opposite; it is organized in a relational and personalized way that resists every general form of categorization, and every general first principle. The relationship is formed on the basis of a mutual willingness to go along with the opacity between midwife and/or doula and the pregnant person and their wishes, rather than demanding transparency and obedience as a prerequisite for a relationship of care, or sacrificing the relationship of care to the first principle of the institution:

The institution of the hospital is so disciplinary. […] This was completely different with my own midwife. She simply came to sit at the table with me and took my medical history on the couch with me. Yes, really, I just thought: this is brilliant. […] I really liked it because we remained in my process, she came to visit me, and I could also show her a bit of who I was and how we were. She saw my kids, she just kind of joined in. I loved it. (Mother 4)

In the quote above, the mother describes that where the obstetric system issues a call to order, severing the relationality and care that was already going on, this midwife does exactly the opposite: she joins the movement and rhythm of already existing care, and blends into it, instead of disrupting it. This testifies to a fundamentally different understanding of what care is, and of what kind of relationality is needed in care. From the perspective of the midwife, this is described as the following:

We were more in dialogue, I left more to the woman, more to herself, I waited more, did less and less. That was the most important: I started doing less and less. I ended my education very assertively, like ‘I’ll do those deliveries’—that was a very normal saying back then. Yes, I was trained to be very assertive, to coach pushing very much from the first moment. And slowly, I unlearned that, I began to see how little you must do. That midwifery is mostly not-doing. (Midwife 2)

Both the midwives, mothers, and doulas, refuse the institutionalization and protocolization of birth, letting birth run its own course within a newly reconstructed relationality of care wherein the mother reappropriates her relationship with her child by birthing herself *and* with a community of care that does not captivate her, but lets her be. It is this relationality, wherein there is no prior principle nor ultimate hierarchy, authority, or goal, that is the primary substance that the undercommons of birth are made of. The independent midwives and doulas in this study see it as their most important job to guard and constitute this relationality; they make sure the emotional safety and freedom it provides keeps on existing:

There are fragile moments in which you can cross someone's borders, I feel that in every fiber of my being. I don't know if I’m important as a person, but it is important that there is always someone with her who is aware of that. (Midwife 11)

Midwives do have to be careful, however, that they make sure that the relationality of midwifery indeed stays anarchic, and that there is not a new first principle or dogma that takes over. Some participants flagged that the resistance to the obstetric institution of certain midwives and doulas tends to be reactionary and dogmatic, highlighting that this can again become a form of care wherein certain values and principles—such as the prioritization of the natural above the technological—of the care worker decide how care should be, rather than it staying truly open and thus be truly liberating. Also, it was noted by some mothers that some midwives and doulas have the desire to care so differently and resist every form of authority, hierarchy and disciplination, that they are regarded by the people who seek their care as dogmatically “soft,” which made the mothers uneasy. Both these critiques were not regarded as a reason not to be cared for by these midwives, however. The participants point out, that the difference with the institution remains that where problems with care are almost impossible to address or change within the obstetric system, in the personal relational forms of care outside of the institutions, one can more easily address and discuss potential problems, due to the individualized and small-scale character of the care offered. But there is an obvious risk here, namely that alternative forms of care develop their own dogmas, rather than dare to stay with the anarchic character of relational care that would be the true alternative.

#### Obstetric abolition

The last subtheme is termed “obstetric abolition.” Midwives and mothers lost faith in the possibility to reform the obstetric institution, which is why they became part of an undercommons of care. Most argued for radically new systems of care rather than for reforming the institution that we have. They could only imagine radical change either outside, or instead of, the obstetric institution—a call for dismantlement rather than reform that can be called abolitionist:

I wish there was no nationalized birth care, […] I wish there were no controlling powers in birth, […] that we do things radically different. (Midwife 1)

Most participants would not give birth themselves in the institution in the way it exists now, if they were to give birth (again). When asked to reimagine birth care in a reimagination exercise in the heterogenous focus groups, most participants reimagined maternity care outside of the institution as we know it today:

Everything just kind of goes on as normal. You know you’re surrounded by your family. The world goes on, while you create this little bubble for birth. It’s definitely centered around the home, away from institutions. There’s this other people present who the person giving birth chooses. If there’s other children in the home and in the family, then they’re around. Birth is something not to be scared off and not to stick away inside a special building. […] It's like everyday life with a quiet, intimate moment at the center. (Doula 5)

[I see] a little hut on a hill. Or a little house on a hill. It doesn’t matter. For me, it stands for freedom. For living outside of systems. And to be able to be born, and die, outside of systems. [..] And then for birth to be something joyful, in which you experience this kind of freedom and autonomy. For there to be less fear and more knowledge of birth itself or of the setting. […] And to be helped by someone that you know, has faith, who is confident and relaxed. (Midwife in Training 1)

According to many participants, only fundamental change could create a different, life-affirmative system of care. An example of fundamentally differently organized care would for instance be if care was not defined by obstetrics, but by the relationality of midwifery:

The moment the whole system would be midwifery, […] then there is, for instance, an obstetrician who comes to a home birth, because that is very important for a woman. Then obstetricians would simply work outside of the institution as a colleague of mine once experienced; a woman could give birth on the birthing stool, with her own midwife and an obstetrician in the corner. (Midwife 11)

Understanding the obstetric institution as a system that reproduces inequality, as almost all participants do, a radical change of the way we care for birth is something that could have great consequences according to most participants. Their vision for what another type of care for birth means, goes beyond the physical event of birth, and is directly related to questions of world-making. This can be regarded as an abolitionist vision, wherein the abolition of something is always related to the abolitionist creation of better worlds:

What you can gain is that you don’t facilitate inequality before someone is even born. That the start in which you are born as a person is more equal. And if you prevent women from being traumatized by childbirth, in whatever way, you indirectly ensure that an entire generation grows up differently and probably happier or healthier for that reason. And if an entire generation grows up better, then that in turn has a lot of effects for the rest of the world. […] I think that’s how you make the world a little better every time. (Midwife 9)

## Discussion: The Undercommons of Childbirth and Their Abolitionist Ethic of Care

Both the expropriation that mothers, midwives, doulas, and midwives in training experience, as well as the carcerality that the participants bring to the fore, are intertwined with the obstetric violence that all participants have experience with. Together, they contribute to the institutionalized separation of a double relationality that is constitutive of maternal subjectivity: (1) the relation between pregnant person and their child; and (2) the relation between the pregnant person and their community of care. According to the participants, obstetric violence should not be understood as a stand-alone problem that is easily circumscribed, but as part of a logic inherent to the obstetric institution that separates relationality, leading to the isolation of the laboring person, through (1) the expropriation of maternal subjectivity, (2) the carcerality of the obstetric institution, characterized by captivity and punishment, and (3) obstetric violence.

Carcerality is understood by Marquis Bey as a characteristic of a system that is “penchant to proliferate capture and expropriation along racist and sexist axes […] via assumed ownership over racialized and/or non-masculinely-gendered subjects, circumscription […], regulation of movement and inhabitation of private space, and extraction of surplus goods and resources (be it labour, sex, sexual labour, time, etc.)” (Bey, 2020). Bey develops further what has been at the heart of institutional critique since Foucault, who has famously shown how most modern institutions are modelled after penitentiary institutions (Foucault, 1977). The description of the obstetric care system as carceral is not new, but stands in a tradition of Ann Oakly's *The Captured Womb: A History of the Medical Care for Pregnant Women* (1984), Adrienne Rich’ *Of Woman Born* (1976), and is in line with what Horn (2023) has recently coined “obstetric carcerality” in her piece *Birthing while Black*: “Handcuffed by a cannula, strapped to a cardiotocograph that monitors my baby's heartbeat, I writhe with pain. The hospital bed physically and mentally shackles me. The aching pierces beyond the body like intense waves crashing through me. This was the prison in which I feared birthing my son.” According to our participants, the obstetric system indeed makes mothers and midwives feel captured, and control and disciplination are effectuated through a fear of punishment. This carcerality, in combination with expropriation and violence, isolates the pregnant person both from their maternal relationality, of which responsibility for the child is one of the defining elements, as well as from their relation with a community of care, constituting the double separation that we have termed “institutionalized separation.”

If we understand obstetric violence to be part of a broader problem of the separation of relationality, then, according to the participants, the project must be to heal and reappropriate these relations. And if it is the case that the obstetric institution is defined by the institutionalized separation of relationality, then the participants rightfully question the possibility of reappropriating relationality within the institution itself. Instead, most take on a, what Marquis Bey (2020, p. 84) calls, “fugitive” approach. Fugitivity is a concept developed in the Black radical tradition and is based on the practice of the maroons wherein formerly enslaved people escaped the institution of slavery and started other communities fully outside the institution. Many Black scholars have expanded their understanding of fugitivity to other current forms of activism, care, and the building of abolitionist worlds, such as Bey's fugitivity when it comes to gender and the gender binary in *Black Trans Feminism* (2022), and Moten and Harney's conceptualization of fugitive planning as the opposite of “policy” when it comes to the institution of the university and the practice of study. Fugitive planning, according to Moten and Harney, is every practice contributing to the liberation of study which became captivated and controlled by the institution that is the university. Together, these alternative practices constitute the undercommons of study, where studying, freely and collectively in sociality, is still possible. Similarly, developing a critique of the institutional policy of obstetrics, our participants attempted to liberate the practice of birth from obstetrics by planning to labor elsewhere—by planning other types of care by tapping into, or further developing, the undercommons of childbirth defined by an anarchic relationality. These undercommons of childbirth refuse the “call to order” of the institution and rebuild care by strengthening the relationality that already exists in homes and communities (Harney & Moten, 2022).

The activist reaction of mothers, midwives, and doulas to the situation they are in, is hence not an attempted reform of the obstetric institution. Instead, they tend to take direct responsibility through caring *otherwise,* expressing their moral convictions through an alternative praxis of care, defined by, what we have termed “anarchic relationality.” As such, the undercommons of childbirth can be understood as expressive of an ethics of care, functioning as Walker's collaborative-expressive ethical model describes, through experiments, experience, and collaboration (Walker, 2007). These undercommons of care are reflexive of the four phases of care that care ethicist Joan Tronto distinguishes, practiced by both midwives *and* mothers: first, they are attentive to their own and each other's needs; second, they take responsibility for them; third, they take care of each other; which is, fourth, fully guided by the responsiveness and feedback of the one they care for, rather than directed by protocols (underscoring the anarchic character of relationality and care). Tronto's four phases of care are fully organized within these relational undercommons of care, outside of the obstetric institution in a way that affirms and reappropriates the relational communality and responsibility of all involved (Tronto, 1993). Interestingly, as such they also constitute Tronto's fifth phase of care, namely “caring with,” that carries the responsibility of making sure that all the four phases of the care cycle above are able to take place. According to Tronto, the organizational repsonsibility of this fifth phase of care constitutes a “caring democracy” (Tronto, 2013). In the case of obstetric violence, however, safe care is no longer guaranteed by the national commons (such as national public healthcare institutions) of the “caring democracy.” Instead, the mothers, midwives, and doulas in this study, make sure *themselves* that this fifth phase is still taken care of, thereby establishing a “caring democracy” underground, or, a caring “undercommons.” The undercommons of childbirth can thus be understood as an *otherworld* wherein responsibility for the four phases of care is taken, but not by the national “commons” of democracy, but by the mutual aid and fugitive planning of mothers, midwives and doulas themselves. It is this fifth phase then, which makes these loose alternative practices truly into an undercommons, that is, a new commons, an *otherworld* of care with abolitionist potential, since they take over the responsibility of the caring democracy. As such, they become the body that replaces the solidarity and trust that connects people to the national community of the caring democracy, through the establishment of an underground communal substitute responsible for, and expressive of, the moral good when it comes to the realization of respectful maternity care and reproductive and birth justice.

These five phases of care in the undercommons, are hence expressive of a specifically *abolitionist* ethic of care, since they become the *substitute* of a caring democracy, thereby constituting a new (under)commons of care, dissenting from the old commons that the caring democracy provided in the form of the obstetric institution. Hence, the undercommoning of the obstetric institution is not only a fruitful ground to reimagine reproductive care and justice anew, but is itself already a form of dismantlement of the obstetric institution. The thick relationality of the undercommons works, as Harney and Moten write, like a “kink” in the cable of the institution, as these networks of care resist, in our case, the access to pregnant people and their institutionalized separation, creating “inaccessible relational refuges of life” (Harney & Moten, 2022, p. 121). These refuges outside the institution are what Foucault did not thematize in his conceptualization of institutions and their discursive influence on the world: the networks of care, relationality, epistemology, and praxis that have always existed and kept on existing outside of the institutions and discursive structures of the state, discursively producing otherworlds next to the hegemonic one, outside of the carcerality of institutional governance. Foucault was able to ruthlessly critique the power that institutions have in shaping our world, but as a post-structuralist rather than decolonial thinker, he did not theorize that there was, after all, an outside to the hegemonic discursivity of modernity in the way that the undercommons shape otherworlds of care, subjectivity, and community, slowly undoing the hegemonic one. Abolition, which has at its objective to dismantle this world and its institutions, has as its aim to “change one thing: everything” (Gilmore, 2022)—through alternative practices of care outside of, and through, hegemonic institutions. The undercommons of childbirth can be understood as one such practice of abolitionist care, as it, indeed, replaces current failures of democratic societies with the horizon of *another* world (da Silva, 2014).

The undercommons of reproductive care must, however, not be misunderstood—especially not by themselves—as a move to pre-modern, pre-obstetric times. Midwifery in white middle class circles too often risks getting lost in a reactionary ideology that is characterized by being anti-modern, as well as anti-technological, anti-gender, anti-trans, anti-abortion, anti-medical, anti-LGBTQ+, and anti-interventionist. According to our study, the abolitionist ethic of care in the undercommons expresses something quite different, and must continue to do so, exactly because of its anarchic character. Since there can be no first principle that functions as a command, there can be no being for or against medicine or technology, or other first principles that structure the relationality of abolitionist care with new dogmas or commands. Instead, the abolitionist care of the undercommons relies on a relation of openness, trust, the affirmation of opacity rather than the demand of transparency, and on personal histories, personal preferences, and solidarity (van der Waal, 2023). The undercommons are not anti- or pre-modern, but they, exactly as the words says, undercommon modernity; they parasite on it, taking what they need and want, strengthening their relational community of care that exists, though only slightly, outside of it in search of abolitionist futures. The abolitionist care of the undercommons is hence by no means a call for the destruction of technology, medical knowledge, or life-saving obstetric care, it is the opposite. Abolition is a call for presence, not absence, as Ruth Wilson Gilmore always says; a call for everything we need in a life-affirming world. In the case of technology and medical intervention, it is a call to dare to think of technology and medicine outside of the obstetric institution, to ask how we can affirm the autonomous anarchic independent undercommons of reproductive care and let our networks flourish with all the technology, medicine, gynecologists, obstetricians, nurses, midwives, doulas, painkillers, and interventions we need. The challenge lies in asking how we will have appropriate anesthesia and C-sections without being captured in obstetric carcerality, isolated through expropriation, and dehumanized through violence. The only question regarding technology and medicine truly fit for the undercommons, is how medical technology could amount to a blossoming of a relationality so thick that we can never be forced (how) to give birth again.

## Conclusion

In this study, we offer an analysis of obstetric violence through a study of the standpoint of the involved actors, such as midwives in training, practicing midwives, doulas, and mothers. We engage with their understanding of obstetric violence and racism, and with the question how they resist. Through putting the standpoint of the participants in dialogue with critical theory, especially with care ethics and abolitionist thought, two main themes are established: (1) “institutionalized separation” with the subtheme's “expropriation,” “carcerality,” and “obstetric violence,” and (2) “undercommoning childbirth” with subthemes “fugitive planning,” “anarchic relationality,” and “obstetric abolition.” Institutionalized separation is understood to be the separation of multiple relations of the pregnant person, i.e., a severance of relationality between mother and child, and from a partner, a community of care, and/or midwives and other birth workers, and a consequent experience of isolation. Obstetric violence is understood to be part of the logic of institutionalized separation, rather than a stand-alone problem. This particular analysis of the problem of obstetric violence determines the participants’ choice of strategy to combat obstetric violence. The second main theme describes this specific strategy of the participants to resist and is coined “undercommoning childbirth.” Undercommoning childbirth is understood as the formation of a network of knowledge, mutual aid, and radical care. The aim of this strategy is to reconstitute or heal the relationality that was broken through institutionalized separation and to resolve the experience of isolation. For, as the participants know, but what is less often recognized in academic research and public policy documents on obstetric violence, is that if a severance of relationality and an expropriation of (relational) autonomy causes obstetric violence, it must be a healing of relationality and a refusal of severing the relationalities that already exist, that will abolish the existence of obstetric violence. Our participants therefore turn to fugitive planning rather than policy in their struggle against obstetric violence. And we believe that their fugitive planning which is constitutive of the undercommons of childbirth, can, in its relation to the obstetric institution (but also beyond it), be understood as expressive of an abolitionist ethic of care.
